# Is the Antiproteinuric Effect of Cyclosporine A Independent of Its Immunosuppressive Function in T Cells?

**DOI:** 10.1155/2012/809456

**Published:** 2012-06-13

**Authors:** Bin Zhang, Wei Shi

**Affiliations:** Department of Nephrology, Guangdong General Hospital, Guangdong Academy of Medical Sciences, Southern Medical University, 106 Zhongshan No. 2 Road, Guangzhou 510080, China

## Abstract

The antiproteinuric effect of cyclosporine A(CsA) has been believed to result from its immunosuppressive effect on the transcription factor NFAT in T cells. However, current evidences supporting this hypothesis are missing. A recent study showed that CsA has a direct antiproteinuric effect on podocytes, suggesting a novel non-immunosuppressive mechanism for CsA's antiproteinuric effect. Conditional NFATc1 activation in podoyctes per se is sufficient to induce proteinuria in mice, indicating that NFAT activation in podocytes is a critical pathogenic molecular event leading to podocyte injury and proteinuria. Meanwhile, evidence showed that TRPC6-mediated Ca^2+^ influx stimulates NFAT-dependent TRPC6 expression. Altogether, these advances in podocyte research indicate that calcineurin-NFAT signal or calcineurin-synaptopodin axis has a direct proteinuric effect on podocytes which raises the possibility of developing specific antiproteinuric drugs that lack the unwanted effects of calcineurin or NFAT inhibition.

## 1. Introduction

Clinically, calcineurin inhibitors (e.g., cyclosporine A, CsA) have been used to reduce proteinuria in focal segmental glomerulosclerosis (FSGS), minimal change disease (MCD), and other proteinuric kidney diseases [1]. T-cell dysfunction is associated with some forms of proteinuria, including a subset of MCD in children. This concept originally stemmed from the so called “Shalhoub hypothesis” that “lipoid nephrosis” is produced by a systemic abnormality of T-cell function [2]. For decades, FSGS was thought to be an immunologic disease resulting from the noxious effect of a lymphokine on the podocyte [3]. This was the primary reason for using CsA as an immunosuppressive drug, to continue to endorse this mechanism of action despite studies demonstrating that calcineurin inhibition reduced proteinuria in nonimmunologic glomerulopathies. Actually, CsA can also reduce proteinuria in human and experimental Alport's syndrome, a nonimmunological disease, raising doubts of this hypothesis [4, 5]. Moreover, although CsA effectively lowered the level of proteinuria in human membranous nephropathy, the study of Ambalavanan et al. showed a more numerous and lager electron-dense immune deposits than before CsA's therapy, indicating that whilst CsA exerts an antiproteinuric effect, the drug does not modify the histologic aggravation of the glomerular lesions [6]. A recent study showed that CsA has a direct antiproteinuric effect on podocytes [7].CsA blocked calcineurin-mediated dephosphorylation of the actin-organizing protein synaptopodin, a podocyte foot process cytoskeletal component, facilitating its degradation by Cathespin L. Mechanistically, this study thus identified a new calcineurin signaling pathway in kidney podocytes and attributed the antiproteinuric effect of CsA to its inhibition of calcineurin-mediated degradation of synaptopodin. Thus, this antiproteinuric effect was shown to be independent of T cells, at least partially. Although arguing against an antiproteinuric role of CsA through the suppression of T cells, this study did not rule out the involvement of NFAT proteins downstream of calcineurin in kidney podocytes. Our results, along with those from Wang et al. [8] and Nijenhuis et al. [9], suggested that conditional NFATc1 activation in podocytes per se is sufficient to induce proteinuria in mice. Thus, both studies provided in vivo evidence that NFAT activation in podocytes may be a critical pathogenic molecular event leading to proteinuria or FSGS. Altogether, these advances in podocyte research indicate that calcineurin-NFAT signal or calcineurin-synaptopodin axis has a direct proteinuric effect on podocytes, and these observations raise the possibility of developing specific antiproteinuric drugs that lack the unwanted effects of calcineurin or NFAT inhibition [10].

## 2. Proteinuria and Its Molecular Mechanisms in Podocyte

Proteinuria, a cardinal sign and a prognostic marker of kidney disease, affects several hundred million people worldwide [12]. Proteinuria is also an independent risk factor for cardiovascular morbidity and mortality. Podocytes, endothelial cells, and the glomerular basement membrane (GBM) constitute the glomerular filtration barrier, a highly specialized structure for selective ultrafiltration. The common denominator in a variety of kidney diseases, including MCD and FSGS, is podocyte injury involving a massive loss of protein in the urine (proteinuria) [13, 14]. Several studies showed that the podocyte has a central role in the development of proteinuria and idiopathic nephrotic syndrome [14]. Effacement of the podocyte foot processes is a common feature of proteinuric diseases [15]. Several pathogenic pathways involved in effacement of the podocyte foot processes and the development of proteinuria have been discovered. Studies in hereditaryproteinuric syndromeshaveuncovered that mutations of podocyte proteins, including *α*-actinin-4 [16], CD2AP [16, 17], nephrin [18], PLCE1 [19], podocin [20], TRPC 6 [21, 22], formin protein INF2 [23], and MYO1E [24] lead to proteinuria, podocyte foot processes effacement and podocyte actin cytoskeleton disruption [14, 25]. Other proteins regulate the podocyte actin cytoskeleton and are important for the glomerular filtration barrier [25]. These proteins include Rho GDIalpha [26, 27], podocalyxin [28], FAT1 [29], Nck1/2 [30], and synaptopodin [31].

TRPC6 is a member of the large transient receptor potential superfamily of nonselective cation channels [32, 33]. Mutations in the gene that encodes TRPC6 have been identified in families with autosomal-dominant FSGS [34–36]. Many calcium-dependent signals, including calcineurin, may be potential targets of TRPC6 activation in podocytes since TRPC6 are involved in the regulation of intracellular calcium concentration in response to the activation of G-protein-coupled receptors and receptor tyrosine kinases. Meanwhile, some TRPC6 mutations found in human beings with FSGS result in increased amplitude and duration of calcium influx into HeK293 cells [21, 22]. TRPC6-deficient mice do not show any obvious renal phenotype [34]. However, transient induction of TRPC6 overexpression by in vivo gene delivery results in proteinuria [33].

The actin-binding protein synaptopodin, which is highly expressed in podocytes [35], is a key regulator of podocyte function since bigenic heterozygosity for synaptopodin and CD2-associated protein results in proteinuria and FSGS [37]. Synaptopodin induces stress fibers by stabilizing the GTPase RhoA [31] and suppresses filopodia by disrupting cell-division cycle-42-insulin receptor substrate p53-Mena signaling complexes [38].

Recently, urokinase receptor (uPAR) and its soluble form (suPAR) have been shown to be involved in the pathogenesis of proteinuria and FSGS [39, 40]. uPAR is a glycosylphosphatidylinositol-anchored protein that has been shown to be a proteinase receptor for urokinase but has also been involved in nonproteolytic pathways, mainly through interactions with other plasma membrane proteins such as integrins [41]. uPAR and *β*3 integrin colocalize in podocytes and thus form a complex with *β*3 integrin, thereby causing the activation of *β*3 integrin. In vivo gene delivery of constitutively active *β*3 integrin is sufficient to induce proteinuria in mice; conversely, inhibition of uPAR expression and *β*3 integrin activation has an antiproteinuric effect [42].

Interestingly, suPAR, a soluble form of uPAR has been identified as a circulating FSGS factor that is elevated in the serum of approximately two-thirds of primary FSGS patients. suPAR-mediated activation of *β*3 integrin on podocyte foot processes may be the mechanism of injury caused by high suPAR blood concentrations [40].

## 3. The Role of Calcineurin Signal in Podocyte Injury and Proteinuria

The calcineurin is ubiquitously expressed in all mammalian tissues and is a Ca^2+^-dependent serine/threonine phosphatase composed of a catalytic subunit, CnA, and a regulatory subunit, CnB [43, 44]. Calcineurin has a wide range of roles in organ development and cellular functions [45, 46], including the regulation of transcription in various renal cells [43, 44].

Clinically, CsA is used to reduce proteinuria in kidney diseases, such as idiopathic nephrotic syndrome (especially FSGS). The immunosuppressive effect of CsA results from inhibition of signaling by the transcription factor NFAT in T cells [47], and this action has also been believed to mediate CsA's antiproteinuric effect. Although the efficacy of CsA was thought to derive from its suppression of NFAT activation in T cells through the inhibition of calcineurin, not all drugs suppressing T-cell activation have protective effects on glomeruli [1]. The study of Faul et al. [7] suggested a novel role for CsA in inhibiting the dephosphorylation of synaptopodin by calcineurin. Faul et al. [7] found that activation of calcineurin in the podocyte is sufficient to cause proteinuria via the degradation of synaptopodin and that CsA blocks the calcineurin-mediated dephosphorylation of synaptopodin, thereby preserving the phosphorylation-dependent synaptopodin-14-3-3b interaction. They also identified synaptopodin as a substrate of PKA and CaMKII. CsA and E64 ameliorate LPS-induced proteinuria by blocking the CatL-mediated degradation of synaptopodin [48–51]. These data unveiled a calcineurin signaling pathway, which is operative in podocytes and contributes to the maintenance of kidney filter function.

Although Faul et al. argued against an antiproteinuric role of CsA through the suppression of T cells, it is possible for the involvement of NFAT proteins downstream of calcineurin in podocyte injury, since the NFAT transcription factors are the most extensively studied calcineurin substrates and the major regulators of transcription in response to Ca^2+^/calcineurin signals [47, 52].

Ca^2+^ signaling through ion channels has recently emerged as a potential modulator of podocyte function, and several Ca^2+^-permeable channels have been identified inpodocytes [53, 54]. Generally, upon activation by increased intracellular Ca^2+^, calcineurin dephosphorylates the NFAT proteins that reside in the cytoplasm in resting cells. This dephosphorylation exposes the concealed nuclear localization signals of the NFAT proteins, leading to the cytoplasm to nucleus translocation of these proteins. In the nucleus, the NFATc proteins form NFAT transcription complex with their nuclear partners to control the transcription of target genes.

TRPC6 mutations were found in families with hereditary FSGS, and TRPC5 and TRPC6 channels are now known as the Ca^2+^ influx pathways for the nonselective, cationic current in podocytes [55]. Mutations in one of these channels,TRPC6, lead to aberrant Ca^2+^ signaling, podocyte dysfunction [21, 22], and Nephrin and Neph1 have been shown to interact with several Ca^2+^ channels, including TRPC6 [11, 56]. Vassiliadis et al. [57] showed that Ca^2+^/calcineurin signals mediated podocyte injury. Inhibition of calcium channels and chelation of extracellular calcium reduced protamine sulfate-induced damage, suggesting that calcium signaling plays a critical role in the initial stages of glomerular injury. Calcineurin inhibitors (FK506 and CsA) inhibited protamine sulfate-mediated barrier changes, indicating that calcium signaling acts, in part, through calcineurin-dependent cleavage of synaptopodin. Meanwhile, mutations in TRPC6 enhance the amplitude and duration of the Ca^2+^ channel current which cause NFAT activation, indicating the activation of the calcineurin-NFAT pathway as a potential mediator of FSGS [58].

Importantly, the study of Wang et al. [8] demonstrated that, in parallel to synaptopodin regulation, there may be an additional pathway from calcineurin to podocyte injury and proteinuria that involves NFAT-mediated regulation of known and novel factors important for podocyte function. To study the role of NFAT signaling in glomerular podocytes, Wang et al. [8] created a system for inducible activation of NFAT signaling in podocytes, in which a Podocin-Cre transgene was used to induce the removal of the transcriptional stop cassette in a ROSA26-rtTA allele only in podocytes [59, 60]. And when the cassette is deleted, the ROSA26 promoter drives the production of reverse tetracycline-controlled transactivator (rtTA) in podocytes. When treated with doxycycline, the doxycycline-rtTA complex binds to the *TetO* sequence of the *TetO-*NFATc*1^*NUC*^* transgene. The mice carrying three alleles (*Podocin-Cre, RO-SA26-rtTA, and TetO-*NFATc*1^*NUC*^*) were referred as mutants. Thus, results from Wang et al. [8] provided in vivo evidence that NFAT activation, either in utero or postdevelopmentally, can lead to podocyte injury and proteinuria, which suggest that activation of NFAT signaling may be a key pathogenic molecular change in podocyte injury and the development of proteinuria.

Using a similar model for conditional NFAT activation in podocytes, Nijenhuis et al. [9] demonstrated that podocyte-specific inducible expression of a constitutively active NFAT mutant increased TRPC6 expression and induced severe proteinuria, and that calcineurin inhibition by CsA downregulated TRPC6 expression and reduced proteinuria. Importantly, this study showed that a deleterious feed-forward mechanism, in which TRPC6-mediated Ca^2+^ influx stimulates NFAT-dependent TRPC6 expression, is involved in angiotensin II (Ang II)-associated podocyte injury. In vitro and in vivo models, Ang II, a key contributor to the pathogenesis of glomerular disease, increases TRPC6 expression in podocytes. The regulation of TRPC6 expression by AngII is dependent on TRPC6-mediated Ca^2+^ influx and the activation of the Ca^2+^-dependent calcineurin/NFAT signaling. Tian et al. [61] showed that Ang II resulted in significant reduction in the abundance of synaptopodin, and gene silencing of TRPC6 resulted in loss of synaptopodin in podocytes in contrast, gene silencing of TRPC5 did not affect synaptopodin abundance. TRPC6-depleted cells treated with CsA restored synaptopodin abundance, suggesting an association between Ca^2+^ influx through TRPC5 and TRPC6 channels and synaptopodin signaling in podocytes. Schlöndorff et al. [58] showed that that all three TRPC6 mutations (P112Q, R895C, and E897K) to enhance channel activity lead to enhanced basal NFAT-mediated transcription in cultured podocytes, which are dependent on channel activity and are dominant when mutants are coexpressed with wild-type TRPC6. Activation of NFAT by TRPC6 mutants is blocked by inhibitors of calcineurin, calmodulin-dependent kinase II, and phosphatidylinositol 3-kinase.

In addition, another study showed that calcineurin induces podocyte apoptosis in a genetic model of type 1 diabetes mellitus (Akita mice). In cultured podocytes, activated NFAT promotes podocyte apoptosis in a calcineurin-dependent fashion, and induction of apoptosis by either angiotensin II or endothelin-1 was blocked by a calcineurin inhibitor (FK506). This induction of apoptosis appears to require NFAT-induced gene transcription [62].

In other cell populations, NFATc1 activation leads to distinctive changes in transcription and cellular behavior [63–65]. Interestingly, Rcan1, Wnt6, and Fzd9 were shown to be upregulated in glomeruli isolated from *NFATc1^ nuc^* transgenic mice, making them potentially direct targets of NFATc1[8]. The upregulation of Rcan1, a known target of NFAT [66, 67], may reflect a potential regulation axis of calcineurin, NFAT, and Rcan1 in podocyte. Moreover, upregulation of Wnt signaling was found to be detrimental to podocytes [68], and Wnt signaling was also shown to be upregulated in *NFATc1^ nuc^* transgenic mice, indicating that the upregulation of Wnt signaling seems to be the pathogenesis of NFAT activation-induced podocyte injury and FSGS.

## 4. Conclusion

**Figure 1:**
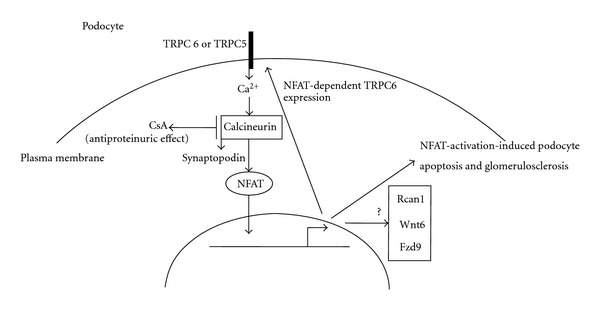
The calcineurin-activation-induced podocyte injury. Upon activation by increased intracellular Ca^2+^, calcineurin dephosphorylates the NFAT proteins and synaptopodin [5, 6]. NFAT activation induces podocyte apoptosis [11] and glomerulosclerosis [6]. TRPC6-mediated Ca^2+^ influx stimulates NFAT-dependent TRPC6 expression [7]. Rcan1, Wnt6, and Fzd9 were shown to be upregulated in glomeruli isolated from *NFATc1^ nuc^* transgenic mice, making them potentially direct targets of NFAT [6].

The antiproteinuric effect of CsA is attributed to its immunosuppressive effect. However, recently published researches showed that the effect of CsA on proteinuria is not dependent on NFAT inhibition in T cells, but rather results from its effects on kidney podocytes, including the stabilization of synaptopodin. Moreover, in vivo evidence from *NFATc1^ nuc^* transgenic mice and Akita mice showed NFAT activation-induced podocyte apoptosis and injury, and NFAT-dependent TRPC6 expression, the mutations of which have been shown to be associated with proteinuria and glomerulosclerosis in human patients. In summary, recently, there is the possibility that CsA might have a coupled nonimmunological antiproteinuric effect: one as demonstrated by Faul et al. on synaptopodin and the podocyte cytoskeleton, another separate one through the inhibition of NFAT as NFAT induces proteinuria and podocyte apoptosis (Figure 1).
